# Impact of 2.45 GHz Microwave Irradiation on the Fruit Fly, *Drosophila melanogaster*

**DOI:** 10.3390/insects11090598

**Published:** 2020-09-04

**Authors:** Aya Yanagawa, Masatoshi Tomaru, Atsushi Kajiwara, Hiroki Nakajima, Elie Desmond-Le Quemener, Jean-Philippe Steyer, Tomohiko Mitani

**Affiliations:** 1 Research Institute for Sustainable Humanosphere, Kyoto University, Uji 611-0011, Japan; mitani.tomohiko.3u@kyoto-u.ac.jp; 2 Department of Drosophila Genomics and Genetic Resources, Kyoto Institute of Technology, Kyoto 616-8354, Japan; tomaru@kit.jp; 3 Nara University of Education, Takabatake-cho, Nara 630-8528, Japan; kajiwara@nara-edu.ac.jp; 4 Department of Molecular Chemistry, Graduate School of Kyoto Institute of Technology, Kyoto 606-8585, Japan; h-nakajima@tezukayama-h.ed.jp; 5 INRAE, Univ Montpellier, LBE, 102 avenue des Etangs, 11100 Narbonne, France; jean-philippe.steyer@inrae.fr

**Keywords:** Electromagnetic waves, Internet of things, living organism, ion-ionizing radiation, ecological influence

## Abstract

**Simple Summary:**

The physiological and behavioral influences of 2.45 GHz microwaves on *Drosophila melanogaster* were examined. This study indicated that there was no concern regarding the thermal effects of microwave irradiation for levels of daily usage if it is traveling waves. However, it still gave non-thermal effects. We detected genotoxicity and behavioral alterations associated with travelling wave irradiation. Electron spin resonance (ESR) revealed that fruit flies possessed paramagnetic substances in the body such as Fe^3+^, Cu^2+^, Mn^2+^, and organic radicals, and the behavioral tests supported the microwave susceptibility of the insects.

**Abstract:**

The physiological and behavioral influences of 2.45 GHz microwaves on *Drosophila melanogaster* were examined. Standing waves transitioned into heat energy effectively when passing through the insect body. On the contrary, travelling waves did not transit into heat energy in the insect body. This indicated that there was no concern regarding the thermal effects of microwave irradiation for levels of daily usage. However, we detected genotoxicity and behavioral alterations associated with travelling wave irradiation, which can be attributed to the non-thermal effects of the waves. Electron spin resonance (ESR) revealed that fruit flies possessed paramagnetic substances in the body such as Fe^3+^, Cu^2+^, Mn^2+^, and organic radicals. The temperature dependent intensities of these paramagnetic substances indicated that females possessed more of the components susceptible to electromagnetic waves than males, and the behavioral tests supported the differences between the sexes.

## 1. Introduction

Electromagnetic fields (EMFs) are used daily in free space and their interaction with living organisms has recently been of concern. For example, 2.45 GHz waves are commonly used in microwave appliances in households [1], as well as for Wi-Fi, Bluetooth, and cordless telephones. Electric fields are known to effect water molecules and have thermal effects on animal bodies, while magnetic fields have non-thermal effects. The effect of EMF on mammals is well-studied [2]. There are several regulations on the use of microwaves based on human specific absorption rate (SAR; W/kg) for wireless applications such as mobile phones. In 1996, the National Academy of Sciences concluded that microwave usage does not affect animal health at the business level of EMF usage because no evidence could be found demonstrating that exposure to EMF causes cancer, neural disasters, or low fecundity, or prevents healthy child growth. However, how it impacts other living organisms such as insects, microbes, or plants is not known [3].

EMFs such as UV light or X-ray are used to control insects and microbes. For instance, X-ray is a widely used treatment for insect male sterilization [4,5]. UV sterilization is also employed in the laboratory clean bench. EMFs such as microwaves used in the industry are strong; thermal and non-thermal effects play an important role in pest control [6,7,8,9]. However, it is not easy to investigate the non-thermal effects on living organisms because the significant damage caused by thermal effects can obscure those caused by non-thermal effects, thus preventing the investigation of non-thermal effects. Evoked electrons under EMFs can affect chemical signaling in cell communications. Paul et al. [10]. reported that EMFs at higher frequencies play an important role in sensing directions, especially in birds. For insects, it is recognized that a chemical compass works with solar and magnetic compasses to determine the direction [11,12]. The ability to perceive Earth’s magnetic field has been reported in many insects including termites, ants, bees, and cockroaches [8,13,14,15,16,17,18,19]. For the fruit fly *Drosophila melanogaster*, Cryptochrome (Cry), the same protein as in birds, can sense Earth’s magnetic field [20,21]. The Cry protein in *Drosophila* is also known to regulate circadian rhythm by sensing UV light [22,23]. Humans emit artificial electromagnetic waves through their industrial and daily social activities. However, it is currently almost unknown whether any living organisms are influenced by these waves. It is thus needed to investigate if artificial EMFs, such as Internet of Things (IoT), can affect other living organisms such as insects, one of the major organisms in our surroundings, to maintain healthy environments along with the quality of life provided by this new technology.

In this study, we exposed *D. melanogaster* to 2.45 GHz microwave irradiation to see how waves affect their physiology and behavior. *D. melanogaster* is a well-established insect as a genetic tool and hence it is used for many biological tests at the same time. It allows multi-approaches to investigate the biological influence of microwaves on this living organism. International Commission on Non-Ionizing Radiation Protection (ICNIRP) put legal ceilings on microwave usage at 1 mW/cm^2^ [24]. The applied power density or power, therefore, was set from 10 μW/cm^2^–100 mW/cm^2^ or 10 W–200 W to cover and exceed this range depending on the capability of irradiation device. To determine the effects, the experiments were design as follows. First, energy transduction patterns in the insect body were monitored. *D. melanogaster* were exposed to microwaves under two different condition; standing waves in a single resonance system and one-way and multiway travelling microwaves. Travelling waves were irradiated to the insects using two devices; single-way microwaves were generated with device 2 and multiway microwaves were irradiated in device 3. Multiway traveling waves were produced by reflections. Next, to learn the physical effects, a genotoxicity test was conducted. As for the collective influence, the influence on behavior was examined by comparing the behavior under microwave irradiation versus no irradiation. Lastly, permittivity and electron spin resonance (ESR) were measured to pursue the mechanism of those influences.

## 2. Materials and Methods

### 2.1. Insects

*D. melanogaster* were maintained on standard cornmeal agar food at 20 °C, and 80% RH. Canton-S (Kyoto Stock Center, Kyoto, Japan DGRC# 105666) was employed in all the experiments, except for in the genotoxicity test. Excluding the genotoxicity tests, three- to five-day-old flies were used in all the experiments. To measure genotoxicity, *mei-9^a^ mei-41^D5^/FM7c; mwh* (Kyoto Stock Center, Kyoto, Japan, DGRC# 109611) and Oregon R(R) (Kyoto Stock Centre, Kyoto, Japan, DGRC# 109612) were used [25].

### 2.2. Direct Exposure to Microwaves under Resonance Condition

*D. melanogaster* were exposed to single-mode microwaves under resonance to determine the impact of industrial microwave irradiation. The microwave system was a solid-state amplifier (R&K GA0827-4754-R) at Kyoto University. This is an analysis and development system for advanced materials and consists of a waveguide cavity with a microwave oscillator (Agilent, MXG wave generator, N5183A), a 3-stub, and a short plunger (Device 1, Supplementary Figure S1) [7]. Microwaves with a frequency of 2.45 GHz are focused by an iris and form a TE103 mode in this cavity. The iris has a 28 mm slit parallel to the direction of the electric field. The EMF was calculated by a finite element method (Software, FEMTET). During microwave irradiation, the profiles of irradiated inflowing microwave power and outward flowing power were continuously monitored with a microwave power meter (Agilent N8485A).

Fruit flies were lightly anesthetized by placing them on ice to stop them from moving. Then, one individual (female ≈ 0.8 mg and male ≈ 0.6 mg) was placed in a glass tube and held still in the arena. Right after it slightly woke up, the insect was exposed to microwaves until it stopped moving for 3 min. Microwave energy absorbance was measured at two different powers with the generator at 10 W (*n* = 11 for each sex).

### 2.3. Direct Exposure to Single Traveling Microwaves under no Reflection

*D. melanogaster*, these insects were exposed to travelling microwaves to determine the influence of ordinal microwave irradiation. The microwave irradiation system was a solid-state amplifier (R&K GA0827-4754-R) at Kyoto University; it consisted of a waveguide cavity with a microwave oscillator (Agilent, MXG wave generator, N5183A) and a dummy load [7]. The 2.45 GHz microwaves irradiate the cavity. The EMF was calculated by a finite element method (Software, FEMTET). Inflowing and outflowing microwave powers were monitored with a microwave power sensor (Agilent N8485A) (Device 2, Supplementary Figure S2).

Fruit flies were lightly anesthetized by placing them on ice to stop them from moving. Then, one individual (female ≈ 0.8 mg, and male ≈ 0.6 mg) was placed in a glass tube and held still in the arena. After the insect wake up, it was exposed to microwaves until it stopped moving for 3 min. The microwave energy absorbance by the insect body was measured at three different generator powers: 50 W (female: *n* = 20, male: *n* = 20), 100 W (female: *n* = 20, male: *n* = 20), and 200 W (female: *n* = 20, male: *n* = 20). To observe the long-term influence, fruit flies were maintained on a standard cornmeal agar food at 20 °C and 80% RH with the combination of 2 males and 4 females in one vial after irradiation.

### 2.4. Direct Exposure to Traveling Microwaves with Reflections Condition

A microwave irradiation system (Sunny Engineering Co., Ltd., MTS03(S), Osaka, Japan) consisting of a semiconductor microwave oscillator and an applicator was used for microwave irradiation. The semiconductor oscillator generates microwave outputs at 10 W, with a single, sharp frequency spectrum at 2.45 GHz (Device 3, Supplementary Figure S3) [7]. This device does not include sensors to monitor inflow/outflow power; instead, it can monitor the temperature inside the applicator and petri dish (DC 3.5 cm) with a thermistor. The material of the petri dish was polystyrene, which interacted with the microwaves in nearly the same manner than air [26]. Thus, heat was efficiently generated in the insect body, rather than the petri dish, by microwave irradiation.

Fruit flies were lightly anesthetized by placing them on ice to stop them from moving. Then, 40 flies were collected in the polystyrene petri dish for each sex (female ≈ 33 mg, male ≈ 24 mg). Irradiation was set to 3 min, and the temperature was measured (*n* = 10).

### 2.5. Genotoxicity Test

The genotoxicity that occurred under microwave irradiation was examined by the wing spot test. This test with *D. melanogaster* indicates the occurrence of mutation [25]. Virgin females of *mei-9^a^ mei-41^D5^/FM7c; mwh* were mated with Oregon R(R) males and allowed to lay eggs for 8 h on a corn meal medium. Three days after egg-laying, the F1 larvae were harvested with a 20 % glucose solution. The larvae floating on the surface of the glucose solution were harvested and washed with distilled water. An equal batch of larvae was placed on a petri dish and irradiated using microwaves for 3 min. To detect a somatic recombination induced by microwave irradiation, the wings of *+/FM7c* females were observed; somatic recombinations were detected as *mwh* mutant spots on the wings (wing spot test). A total of 60 flies were analyzed at each irradiation power.

The microwave power was adjusted in two groups by using microwave devices. For the first group, a portable microwave generator (Device 4, Supplementary Figure S4) was used. Microwaves from the device 4 is possible to adjust its power up 100 mW/cm^2^ at the sample point. Seven arenas were made in the experiment box. *D. melanogaster* larvae were irradiated at 100 μW/cm^2^ (−18.5 dBm at generator) and 100 mW/cm^2^ (−3.3 dBm at generator) at the irradiation surface by using Device 4. Depending on the location of the arena, the power strength varied: 288.8 ± 1.11 μW/cm^2^ for 100 μW/cm^2^ and 78.8 ± 14.64 mW/cm^2^ for 100 mW/cm^2^. Other irradiations were conducted using Device 2 at a power of 10 W and 100 W of the wave generator (Supplementary Figure S2). Device 2 controls its power at the generator, and it employed for this test since it can produce high-power microwaves. The negative control was with no irradiation, and the positive control was with 100 W irradiation from a microwave appliance (National NE-EH22, Japan). In addition, to confirm the relevance of this approach to detect genotoxicity, UV light was irradiated on the larvae for comparison. UV light is non-ionizing radiation, but biologically toxic

### 2.6. Behavior

*Drosophila* can sense Earth’s magnetic field by the Cry in their eyes [20,21]. Insect perception of electromagnetic waves were examined by the comparison of behavior under impressing or irradiating electromagnetic fields or waves, and the behavioral change were reported in some behavior such as walking or turning [6,14], respectively. To observe if there was any change in the insect’s naïve behavior, a ferret behavior was observed in a simple shelter test in a chamber box with microwave irradiation (Device 4, Supplementary Figure S4). This is simply to see if *D. melanogaster* shows repellency to microwaves and tried to stay inside of a shelter covered with aluminum foil during the irradiation. Seven arenas were made in the experimental box. Approximately ten flies were anesthetized by placing them on ice; they were then quickly put into a blue tip. The actuate side of the blue tip was cut to a size such that only one fly could get through. After all the flies were out of the blue tip, the other side of the tip was closed with a cotton ball. Two types of blue tip were prepared: one was covered with a dark-colored paper, and the other was covered with aluminum foil. Microwaves are supposed to be able to pass through paper but not through aluminum foil. Each blue tip was fixed on the bottom of a 9 cm polystyrene petri dish and covered with a lid. Flies were irradiated under 10 µ = μW/cm^2^ (−38.5 dBm at generator) and 100 mW/cm^2^ (−3.3 dBm at generator) microwaves (*n* = 10). Depending on the location of the arena, the power strength varied: 13.8 ± 3.25 μW/cm^2^ for 10 μW/cm^2^ and 78.8 ± 14.64 mW/cm^2^ for 100 mW/cm^2^. This experiment was carried out in the microwave irradiation box, and the flies were left for 30 min or 3 h. The controls were obtained under no irradiation for each sample, and the preference index (PI) was calculated by the number of flies coming out of the blue tip.

PI = (N_out_ − N_in_)/(N_out_ + N _in_) (1)

where N_out_ is the number of flies that came out of the blue tip and N_in_ represents the number of flies that stayed inside the blue tip. The PIs were normalized by the PIs of each control condition to set the control results as PI = 0. The experiments were conducted in darkness without any olfactory/gustatory cues. Consequently, the critical environmental cue for the flies to use should be an irradiated microwave. Because the flies were placed in a blue tip that only had one entrance, the efficacy of their entrance search will reflect their sensitivity to microwaves. The experiment room was maintained at 25 °C. Females and males were tested separately and 1600 flies were used.

### 2.7. Paramagnetic Components

ESR detects the presence of unpaired electrons and visualizes the free radicals in the substance directly and specifically. The spectra indicate the paramagnetic substance while the g value of a material indicates the magnetic dipole component.

The ESR spectra of the radicals were recorded on a JEOL JES RE-2X spectrometer operating in the X-band, using a field modulation value of 100 kHz and a microwave power of 1 mW. The TE011 mode cavity was used. Temperature was controlled using a JEOL DVT2, a variable temperature accessory. ESR measurements were performed at 27, −50, −100, and −180 °C. Spectroscopic simulations were conducted with the JEOL IPRIT data analysis system (version 6.4, JOEL Ltd., Japan) and the mass spectra were analyzed. In addition, a portable microwave irradiation system was set up, from which the ESR spectrum was recorded, also under 2.45 GHz microwaves. It was placed on a mobile rack and consisted of a phased array antenna with circular-polarized antenna elements, a 2.45 GHz microwave semiconductor generator and amplifiers (total maximum power of 50 W) with a computerized beam control unit. Irradiation powers of −38.5 dBm (or approximately 24.3 μW/cm^2^) and −3.3 dBm (or approximately 73.0 mW/cm^2^) were used (Device 5, Supplementary Figure S5).

The fruit flies were first lightly anesthetized by placing them on ice and then transferred to the sample holder (JOEL DATUM ESR No:193 #422000281).

### 2.8. Permittivity

The design of the microwave reactor is influenced by the temperature dependence of permittivity because permittivity affects the characteristic impedance. Therefore, the permittivity of *D. melanogaster* was measured at 23 ± 3 °C using the method proposed by Nelson et al. [27]. For this measurement, females and males were collected together in a vial. Then, the penetration depth in an insect body was calculated with obtained permittivity [28].

The relative complex permittivity, dielectric constant and dielectric loss factor were measured with a Keysight N1501A open-ended coaxial-line probe (N1501A-102, Keysight, USA) and an N5242A network analyzer (Agilent technologies, Japan) (*n* = 10). Insects for the measurements were collected in a polystyrene vial (4-1024-03 As One, Japan). As water is an important factor influencing permittivity, the moisture content of the insects was determined by drying for 72 h at 60 °C. Fruit flies were transferred into a pre-weighed paper bag and measured before and after drying.

### 2.9. Statistics

The measured values/results were compared between those from irradiating treatments and controls. Dunnett test was applied to see the energy transduction patterns, chi-square test was employed for the numbers of wing spots to see the mutation rate and Mann–Whitney test was used to examine the behavioral difference. For other statistics, Wilcoxon test was applied. Except the genotoxicity test, which followed the established common protocol, non-parametric tests were applied to cover the sample size. JMP 10.0 software, (SAS Inc, NC, USA) was used for all analysis.

## 3. Results

### 3.1. Effect of Standing Waves under Resonance Condition

**Figure 1:**
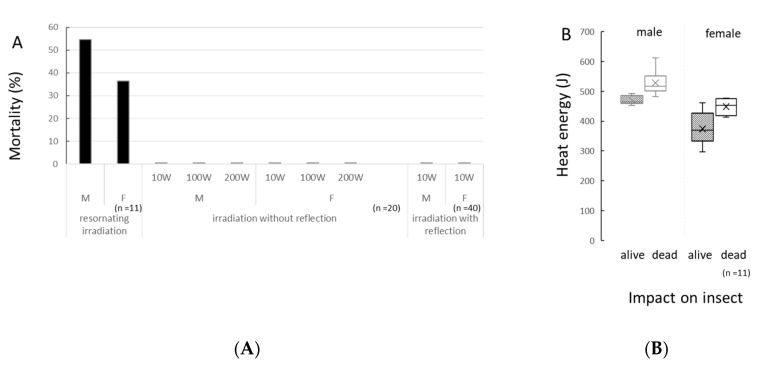
Effect of different energy transduction patterns on the *Drosophila* body under three irradiation conditions. (**A**) Mortality of adult flies under three different irradiation conditions; (**B**) Energy absorbed by an insect body, which was absorbed until the sample insect stopped moving under irradiation with resonance waves (*n* = 11). The horizontal bar indicates the insect status one day after irradiation.

*D. melanogaster* were exposed to microwaves under resonance with single-mode microwaves. Microwave energy converted to heat energy in the insect body. As a result, 90–95% of the microwave energy was constantly absorbed by the fly body as heat energy. Microwaves were irradiated to one sample insect. Irradiation was stopped if the insect did not wake up in 3 s, and they were checked at 10 s intervals. Most sample insects stopped moving in 1 min. Approximately 55% males and 36% females died after exposure (Figure 1A, *n* = 11 for each sex). Female flies died after approximately 47.3 s and male flies after 58.6 s. A female fly died after absorbing an average of 449.0 J of heat energy from the microwave. In comparison, a male fly died by absorbing an average of 528.1 J of energy. Males seemed to be more tolerant to microwave irradiation than females. The lethal threshold of heat energy absorbance seemed to lie between 374.0–449.0 J for females and 471.0–528.1 J for males (Figure 1B, *n* = 11 for each sex).

### 3.2. Effects of Single Way Travelling Microwaves

*D. melanogaster* were irradiated in the device by travelling microwaves, which moved in a straight direction and did not reflect at the side of the device. Microwave power was not converted to heat energy. The monitors indicated that 0–1.5%: (input energy − output energy)/input energy × 100, of microwave energy was absorbed by the sample body. No sample insect died after microwave irradiation for 3 min at powers of 50, 100, and 200 W (Figure 1A, *n* = 20 for each sex at each power). However, an energy transit occurred because generally it was 0 J, but a maximum of 2160 J microwave energy was lost after it passed through an insect body: (input energy − output energy)/input energy × time (s) (Supplementary Figure S6A). Meanwhile, no death after irradiation indicated that the energy from the microwave was not absorbed by the *Drosophila* body. There were no significant differences in the number of pupae from these irradiated sample flies (Wilcoxon test, Supplementary Figure S7).

### 3.3. Effects of Multiway Travelling Microwaves

By using a device that allowed reflections, microwave power was converted to heat energy through the insect body, but with low efficiency. No insect died after 3 min irradiation (Figure 1A, *n* = 40 for each sex). Microwave energy converted to heat energy under this irradiation condition. Similar to irradiation without reflection, a minimum of 262.3 J to a maximum of 2967.0 J of microwave energy was lost after passing through an insect body (Figure S6B). Under this condition, some of the microwave energy seemed to be absorbed by the insect body as heat energy because an increase in body temperature was observed; thus, the heat absorption was calculated. Because the increase was not stable during the initial moment of irradiation, an identity equation for the energy transition was obtained with the data ranging from 20–60 s over the irradiation period (*n* =10) (Supplementary Figure S6B,C). The temperature of the chamber with female flies in a petri dish increased with the equality Tc = 0.048 ± 0.009 s, and that of the empty chamber (control) increased with the equality Tc = 0.024 ± 0.007 s. Hence, the estimation of the energy absorbance rate of the female body is Tt = 0.024 s, an increase of Tc = 0.030 ± 0.007 s was obtained for male flies, and an increase of Tc = 0.005 ± 0.001 s for the empty chamber (control). Hence, the estimation of the energy absorbance rate of the male body is Tt = 0.025 s. (Tc: increase of chamber temperature, s: second, Tt: increase of insect body temperature).

The heat-energy absorption of the *D. melanogaster* body at 2.45 GHz was estimated by the specific heat of water 4180 J/(kg·K) and the water content of the *Drosophila* body (70%).

Female:

Power absorbance P_B_ = [0.033 (g) × 4.180 (specific heat of water) × 0.7 (body water content) × 0.972 (temperature increase for 40 s)]/40 (s) = 0.0023 W = 0.0023 J/s (2)

Absorbance rate (%) = 100 (P_B_/P_A_) = 100 (0.0023/10) = 0.023% (3)

Male:

Power absorbance P_B_ = [0.024 (g) × 4.180 (specific heat of water) × 0.7 (body water content) × 0.994 (temperature increase for 40 s)]/40 (s) = 0.0017 W = 0.0017 J/s (4)

Absorbance rate (%) = 100 (P_B_/P_A_) = 100 (0.0017/10) = 0.017% (5)

(P_A_: irradiation power 10W, P_B_: Power absorbance)

### 3.4. Genotoxicity

**Figure 2:**
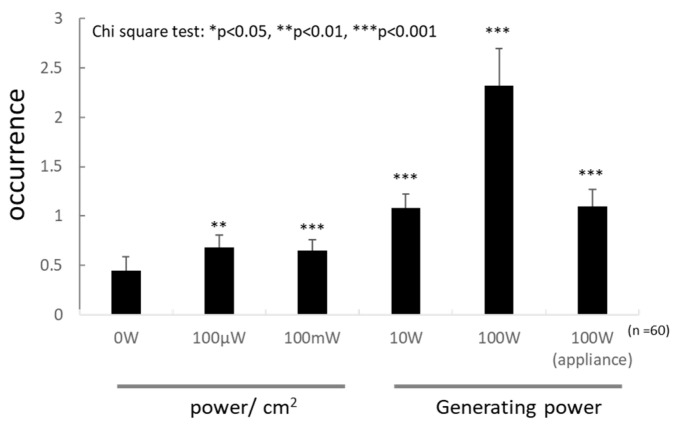
Genotoxicity detected in the wing spot tests by 3 min microwave irradiation. Irradiation powers from 0–100 mW/cm^2^ were set by the insect body surface, which was measured in the middle of an experiment arena in Device 4. Powers ranging from 10 to 100 W were used for the generator of Device 2. Positive control, i.e., 100 W (appliance) irradiation, was also set for the generator of the microwave appliance. Asterisks indicate the significance from negative controls detected in Chi-square test. *: *p* < 0.05, **: *p* < 0.01, ***: *p* < 0.001.

The test was conducted according to the protocol by Fujikawa [25]. Larvae were exposed to microwave irradiation, and the phenotype and number of wing spots were checked after the eclosion. The phenotypes reflect dsDNA breaks, and the number of wing spots reflects the genotoxicity. The recovered F1 flies were classified into the following three classes of phenotypes and were scored: DNA repair-proficient females (F), DNA repair-defective males (M1), and DNA repair-proficient males (M2). Large and small spots were observed on the wings. The number of spots were pooled because most spots were small. The DNA repair-defective male (M1) was rarely detected in the F1 flies. However, the number of spots on the wing increased with the irradiation power (Figure 2, *n* = 60 for each power). Interestingly, the number of spots after 100 W irradiation of the ordinal microwave appliance was much smaller than that after the equal-power microwave irradiation of the lab wave generator. No larva became an adult after the UV irradiation.

### 3.5. Behavior

*D. melanogaster* have Cry proteins, which react to the magnetic field in visual perceptions [20,21]. Hence, the behavioral alteration by travelling wave irradiation was examined. The results support that the insect might perceive microwaves as environmental information. The significance of the total data was apparent for the parameters of sex (female/male: x^2^ = 11.73, *p* < 0.01, df = 160 Kruskal–Wallis test) and irradiation period (30 min/3 h: x^2^ = 29.59, *p* < 0.01, df = 160, Kruskal–Wallis test). Hence, the data were not pooled for further analysis. 

**Figure 3:**
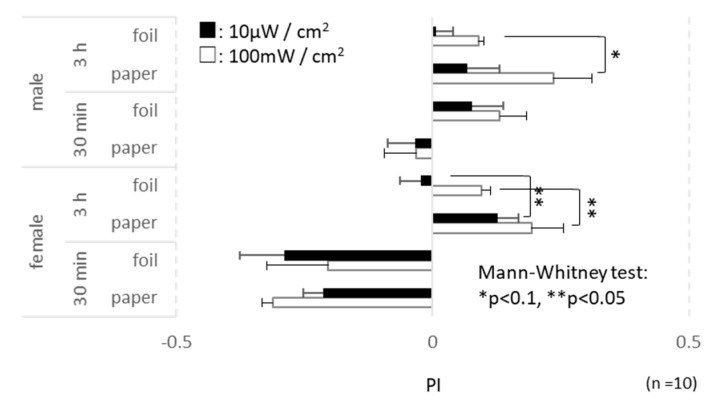
Performance index (PI) of flies coming out of the blue tips after 30 min/3 h microwave irradiation in Device 4. The vertical bar indicates the test conditions, and the horizontal bar indicates the preference index (PI). A high PI score indicates the intensity of the ferret behavior, which means that more flies found the exit of the blue tip. Asterisks indicate the *p* value of the Mann-Whitney test. *: *p* < 0.1, **: *p* < 0.05. *n* = 10 for each sex.

More flies were generally outside after 3 h of irradiation. The number of males outside the blue tip slightly differed between the paper and the foil wraps under 100 mW/cm^2^ irradiation (x^2^ = 2.77, *p* = 0.09, df = 10 at 3 h irradiation, Mann–Whitney test, Figure 3). Males seemed to be more active than females; however, ferret behavior was enhanced in females by microwave irradiation. More females enclosed in the paper wrapped blue tip found the exit hole compared with those wrapped with aluminum foil (10 μW/cm^2^: x^2^ = 5.18, *p* = 0.02, df = 10 and 100 mW/cm^2^: x^2^ = 4.24, *p* = 0.04, df = 10 at 3 h irradiation, Mann–Whitney test, Figure 3). The microwave power also affected the ferret behavior of fruit flies. Under 100 mW/cm^2^ microwave irradiation, more females were inside after 30 min (paper: x^2^ = 3.91, *p* = 0.05, df = 10, Mann–Whitney test, Figure 3), and more females and males were outside after 3 h (females in foil: x^2^ = 4.29, *p* = 0.04, males in foil: x^2^ = 5.13, *p* = 0.02, df = 10, Mann–Whitney test, Figure 3).

### 3.6. ESR Measurements

**Figure 4:**
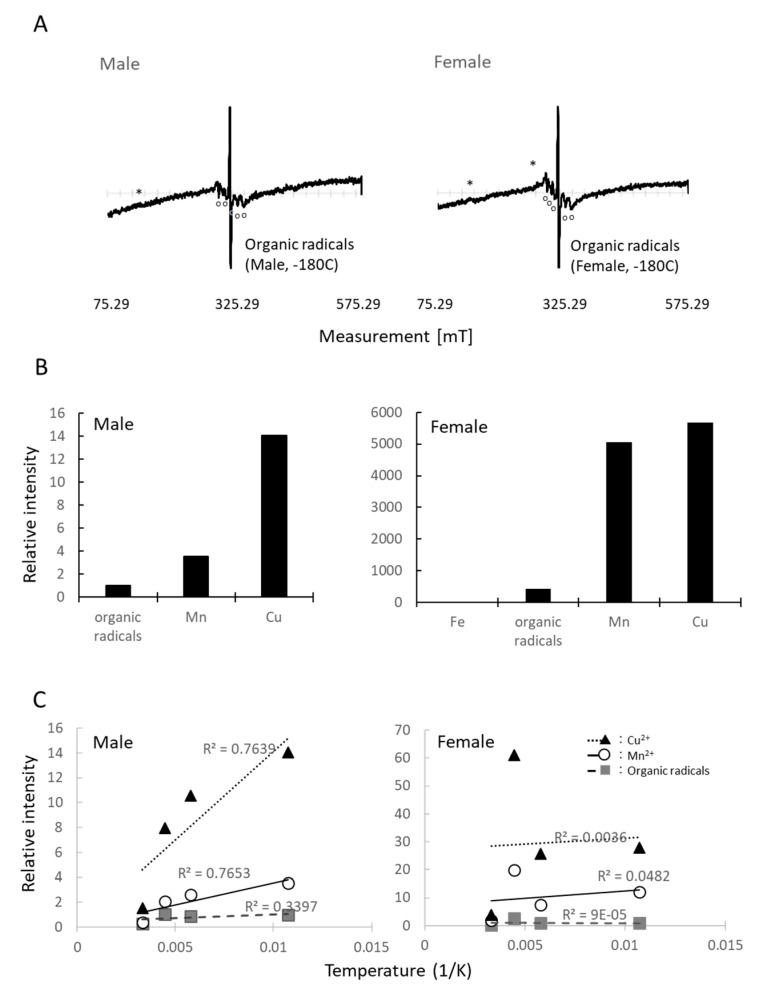
ESR spectra of the fruit flies, *D. melanogaster*. (**A**) ESR spectrum of the fruit flies at −180 °C (male 43.2 mg, female 80.3 mg); (**B**) Relative intensity of paramagnetic substances when organic radicals were set as standard (=1) in males with the intensity at −100 °C, and Fe^3+^ (=1) in females at −180 °C to compare with other paramagnetic substance es. Spin intensity of Fe^3+^ in males was too small to measure; (**C**) Interactions with microwaves suggested by temperature-dependent ESR spectrum. If the relative intensity increased proportionally, it suggests that there was no interaction with microwaves. The intensity at −180 °C was set as standard (=1) for each paramagnetic substance to compare the intensity with other temperatures. K: temperature in Kelvin. * indicates the spectrum of iron (Fe^3+^). ○ indicates the spectrum of manganese (Mn^2+^). The S-shaped curve over the Mn spectra indicates the presence of copper (Cu^2+^). The *g* value shows the dimensionless quantity that characterizes the magnetic moment and angular momentum of a composite particle, particle, or nucleus.

**Figure 5:**
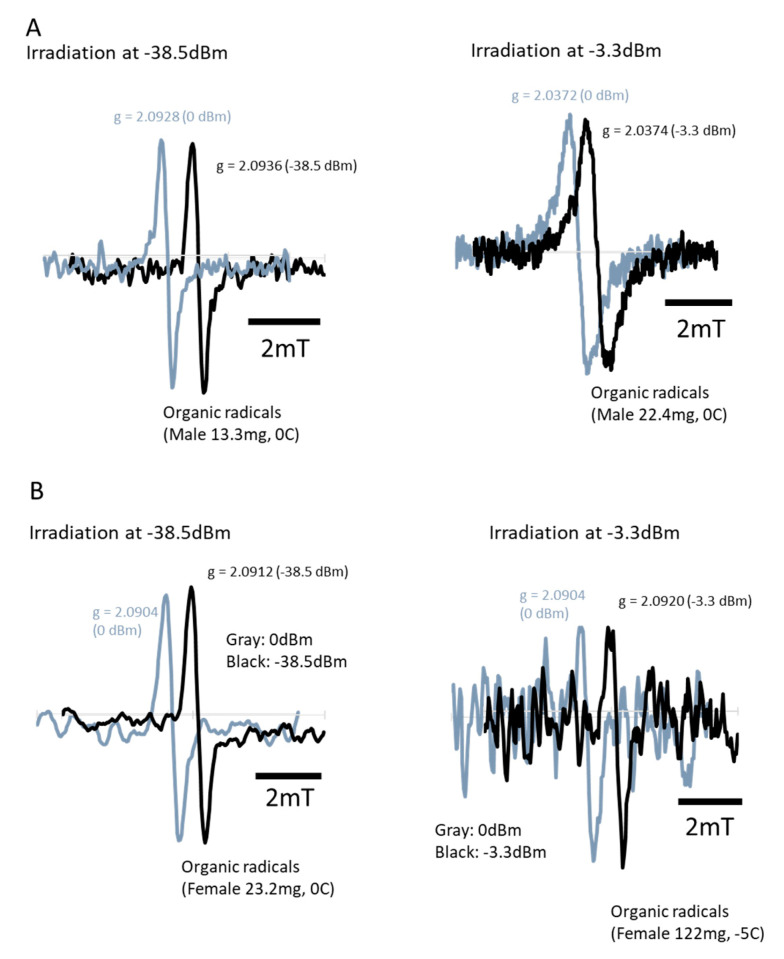
ESR spectra of organic radicals in the fruit flies, *D. melanogaster*. (**A**) ESR spectra of organic radicals in male fruit flies, *D. melanogaster* showing a decrease of free radicals under micro wave exposure of 2.45 GHz at −38.5 dBm, −3.3 dBm (black), and 0 dBm (control, no irradiation, grey); (**B**) ESR spectra of organic radicals in female fruit flies showing an increase under microwave exposure of 2.45 GHz at −38.5 dBm, −3.3 dBm (black), and 0 dBm (control, no irradiation, grey).

Figure 4 shows the ESR spectrum of fruit fly *D. melanogaster*. The ESR revealed that the fruit flies contain several paramagnetic substances in their bodies, such as Fe^3+^, Cu^2+^, Mn^2+^, and organic radicals (Figure 4A, male: 43.2 mg, female: 80.3 mg). The spin intensity of Fe^3+^ was too small to be obtained; however, the relative intensity suggested that *Drosophila* females contained more Mn^2+^ than males (Figure 4B). The temperature-dependent signal intensity indicated the interaction of these paramagnetic substances with other molecules. If the temperature-dependent signal intensity showed a proportionate increase, it would indicate that the substance existed independently in the *D. melanogaster* body. If it combined with other molecules, it would indicate that the interaction between these molecules changed the pattern. Hence, if the temperature-dependent intensity did not show a proportional increase, it suggested that some proteins or peptides combined with the substance in the microwave-sensitive structure. The temperature-dependent signal intensity showed that females possess more paramagnetic compounds, which combine with other molecules. The conditions of the paramagnetic components are different in females and males for organic compounds, Mn^2+^_,_ and especially Cu^2+^ (Figure 4C). Notably, the ESR spectrum during an additional microwave irradiation of 2.45 GHz revealed that organic radicals decreased by −3.3 dBm (Figure 4B,C), with a relative intensity 0.964 and 0.929 for males and females, respectively. However, it increased by −38.5 dBm microwave irradiation (Figure 5A,B), with the relative of 1.113 and 1.085 for males and females, respectively.

### 3.7. Permittivity

Females and males were mixed for the permittivity measurement because it required a large number of insects. Permittivity was varied depending on the density because of the amount of air in the vials; therefore, it was adjusted with the calculation as done in Nelson et al. [27], which can adjust the air influence in the vial. The microwave permittivity at 2.45 GHz at 23 ± 3 °C was 4.2 (measured permittivity: 4.57, Supplementary Figure S8, amount of fruit flies: 1.5 g). The water content of the sample insects was 71.8%.

The average permittivity and Tan (σ) at 2.45 GHz (*n* = 10) from the above measurement, without the offset process, were considered for this calculation.

(6) $$Penetration\;depth=\frac{c}{2\pi f{\left(2{\;\varepsilon \;\prime }_{r}\right)}^{\frac{1}{2}}}{\left\{{\left[1+{\left(\frac{{\;\varepsilon \;\prime \prime }_{r}}{{\;\varepsilon \;\prime }_{r}}\right)}^{2}\right]}^{\frac{1}{2}}-1\right\}}^{-\frac{1}{2}}\;=\;[2.998\;\times \;10^{8}]/2\surd 2\pi \;\times \;2.45\;\times \;10^{9}\;\{4.571[\surd 1\;+\;(0.600/4.571\;{)}^{2}\;-\;1]{\}}^{1/2}\;=6.95\mathrm{cm}$$

(f: frequency, ε_r_′: insect relative permittivity without Nelson’s offset calculation [27], ε″_r_/ε′_r_: dielectric loss)

## 4. Discussion

This study demonstrated the non-thermal effects of microwaves in a living organism, *D. melanogaster.* Although travelling waves did not kill the insects, several physiological effects were detected. First, the energy absorption of the insect body was measured under three different irradiation systems. Under the resonance condition of standing waves, microwave energy converted to heat energy when passing through the insect body, and the sample insects died immediately. However, no insect died under the influence of travelling waves. For traveling waves, we detected genotoxicity owing to irradiation and a behavioral change during irradiation. The permittivity of the fruit fly is 4.2, which is probably insufficient for them to maintain some electric energy inside their body. ESR revealed that flies contain several paramagnetic substances in their bodies, such as Fe^3+^, Cu^2+^, Mn^2+^, and organic radicals. Then, the intensity of organic radicals was varied by the power of microwave irradiation.

We confirmed that traveling waves at the daily usage level in open space did not have any lethal effects on the flies because of the lack of thermal effects from the waves. In general, traveling microwaves seemed to simply penetrate the insect body. This is probably because microwave energy was radiant, and radiation did not heat the sample body. In addition, the body size of the flies was too small to act as a container of electrons, which prevented the microwaves from converting to heat [29]. Under wave irradiation from many directions, the reflected waves comprised standing waves partially in the arena; therefore, some energy converted to heat energy. However, permittivity at 2.45 GHz was 4.2, which is similar to that of other insects [30,31]. The permittivity indicates the ability of a material to hold electrons. The permittivity of *D. melanogaster* indicated that the body of this insect had a mild capacity. The following ESR measurement supported the influence of microwave irradiation; it clearly showed the non-thermal effects of microwaves, i.e., exposure to traveling waves induced changes in the quantity of organic radicals (Figure 5A,B). Because the change was different depending on the irradiation power, it may indicate that the microwave irradiation changed the electric conditions of some molecules. The g value only indicated the magnetic dipole component. Its shift implies that the status of a magnetic moment and angular momentum in the insect body changed because of microwave irradiation. In a previous work, the g value shifted clearly when we irradiated termites [7], but it was not the case here with *D. melanogaster* (Figure 3). For clarifying the reaction of organic radicals, molecular-level approaches on cell conditions, along with a survey of cell communications, would be helpful. For humans, medical healing techniques such as microwave coagulation therapy or hyperthermia treatment are utilized to kill cancer cells using the thermal effects of microwaves [32,33]. Asano et al. [26,34] applied weak microwaves to a mixed culture of healthy and cancerous cells and successfully killed only the cancer cells, which is likely the result of the non-thermal effects. Without a microwave, Huizen et al. [35] demonstrated the changes in new tissue formation by irradiation with a weak magnetic field. Both studies indicate the non-thermal effects of electromagnetic fields on cell signaling pathways.

It is said that *D. melanogaster* are good at sensing Earth’s magnetic field and show naïve avoidance of the field [20]. The Cry protein of *D. melanogaster*, a blue light-dependent regulator, is the input of the circadian feedback loop. No photolyase activity has been reported for cyclobutane pyrimidine dimers or 6–4 photoproducts; however, this protein is known to contribute to sensing Earth’s magnetic field in *D. melanogaster* [20,21]. This Cry activation is intermediated by radicals [10,36]. Hoang et al. [36] reported that a Cry protein subfamily, which does not possess the light response, also showed photoreaction. The regulation of Cry expression by light suggests its role in photoreception for locomotor activity rhythms [20,21]. If flies visually perceive microwaves owing to cryptochrome, they would see the irradiation clearer in the paper shelter, compared with the aluminium foil shelter. Because 100 mW/cm^2^ is a 10,000 times stronger wave irradiation, it is possible that 100 mW/cm^2^ irradiation could penetrate the thin aluminum foil cover but not 10 μW/cm^2^, which is approximately the natural level. It is possible that there was a slight difference in visual clearness caused by the irradiation power in the foil shelter. In addition, because the light adjustment of the Cry protein is variable and can change in 2 h even under weak irradiation [23], the differences for the sexes at each time interval can simply reflect the adjusting time of this protein or other unknown magnetic receptors. In addition, some other papers have reported that there is a phenomenon called microwave hearing in mammals [37]. As the sequence and function of Cry proteins seem to be shared with many living species, including insects and plants [36,38], the behavioral difference could be due to auditory perception, not visual. Thus far, behavioral alterations by wave irradiation have been reported also in mammals [39] but the reason is still unknown. ESR demonstrated that fruit flies possess paramagnetic substances Fe^3+^, Cu^2+^, Mn^2+^; however, the molecular form differs in males and females. It also indicated that *Drosophila* females are more sensitive to microwave irradiation than males. Further study is required to clarify this behavioral alteration and the difference in the responses of both sexes to microwaves.

Two facts indicated the ecological influence of our daily usage of microwaves: the wing spot test, which revealed that the irradiation of traveling waves caused genotoxicity from 3 min microwave irradiation of 100 μW/cm^2^, and the ESR results, which indicated that a −38.5 dBm irradiation (about 24.3 μW/cm^2^) induced an increase of free radicals in the insect body. Both exposures are powers under regulation. In Japan, Ministry of internal affairs and communications (MIC) prescribes the power of 2.45 GHz waves for the human health as average 1 mW/cm^2^ and max 2 mW/cm^2^ for less than 6 min in Radio Act 2017, appendix 2-3-2. This regulation follows ICNIRP. International agency for research on cancer (IARC) classified non-ionizing waves, including microwaves, in Group 2B: the agent is possibly carcinogenic to humans, whose cancer risk is the same as caffeine but less than coffee [2]. The genotoxicity of *D. melanogaster* is also known to be induced by caffeine ingestion [40]. Regardless, the different results from the 100 W irradiation in Device 2 and the microwave appliance are interesting. The occurrence of genotoxicity dropped in the appliance, which is good because it is more important and related to daily life. No effect was detected on fecundity in our test, while in *D. melanogaster* females, Gonet et al. [41] reported egg reduction by a 50 Hz magnetic field. Panagopoulos [42] demonstrated the influence of microwaves from mobile telephone radiation on the ovary, and hence reproduction ability [43,44,45], and Kesari et al. [46] warned the risk of sperm decrease of males using the Wister rat under mobile-phone level radiation. The genes related to propagation could be involved in free radical reactions detected by ESR. Considering the remarkable development of this technique, it is important to conduct a further study on the effect of artificial EMF on many other living organisms.

The non-thermal effects of microwaves have been discussed in both non-organic and organic materials [47,48], respectively. This study demonstrated that artificial microwave usage in our daily life certainly effects surrounding animals. IoT technologies will improve our quality of life and solve many problems in this modern world, making it profitable. However, we must also consider their side-effects.

## 5. Conclusions

This study demonstrated the non-thermal effects of microwaves in a living organism, *D. melanogaster.* The energy absorption of the insect body was measured under three different irradiation systems. Under the resonance condition of standing waves, microwave energy converted to heat energy when passing through the insect body, while no insect died under the influence of travelling waves. Instead under traveling waves, genotoxicity owing to irradiation and a behavioral change during irradiation were demonstrated by the insects. The permittivity of the fruit fly is 4.2 and flies contain several paramagnetic substances in their bodies, such as Fe^3+^, Cu^2+^, Mn^2+^, and organic radicals.

## Supplementary Materials

The following are available online at https://www.mdpi.com/2075-4450/11/9/598/s1, Figure S1: Microwave irradiation, Device 1, to create the resonance condition. Figure S2: One-way travelling wave irradiation, Device 2, without reflection. Figure S3: Multiway travelling wave irradiation, Device 3, with reflection. Figure S4: Traveling wave irradiation, Device 4, for the behavioral test. Figure S5: Travelling wave irradiation, Device 5, to give the additional irradiation during ESR recording. Figure S6: Energy absorption of insect body under 3 min irradiation with straight forward travelling waves. Figure S7: Effects of straightforward traveling waves on *Drosophila* fecundity. Figure S8: Permittivity of the fruit flies, *D. melanogaster*.
