# Supplementary material for: Impact of 2.45 GHz Microwave Irradiation on the Fruit Fly, Drosophila melanogaster
Source: Insects. 2020 Sep 4;11(9):598. doi: 10.3390/insects11090598 (PMC7564283; doi:10.3390/insects11090598)
Supplement: Supplementary file 1 [file insects-11-00598-s001.pdf]

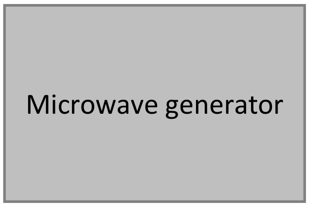

Figure S1. Microwave irradiation, Device 1, to create the resonance condition. Blue arrows indicate the standing microwave route. The grey star indicates the sample insects.

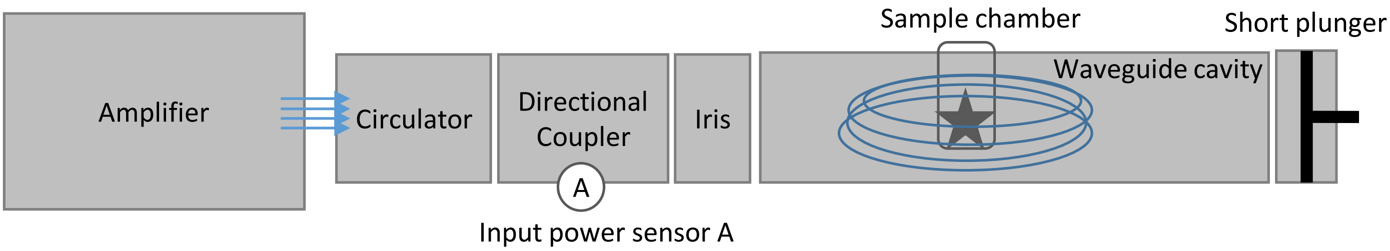

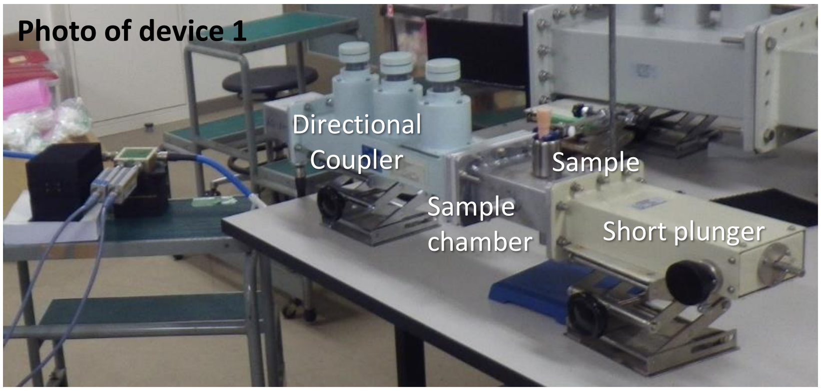

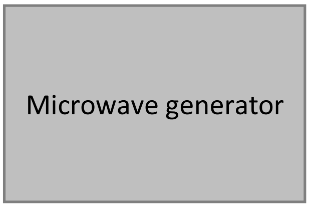

Figure S2. One-way travelling wave irradiation, Device 2, without reflection. Blue arrows indicate the microwave route. The grey star indicates the sample insects.

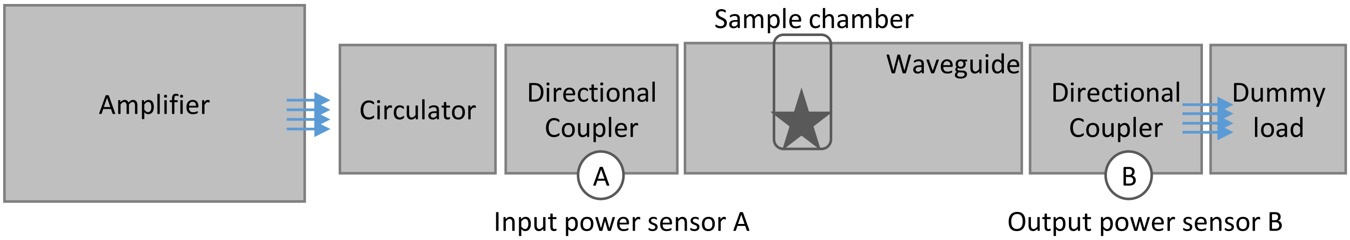

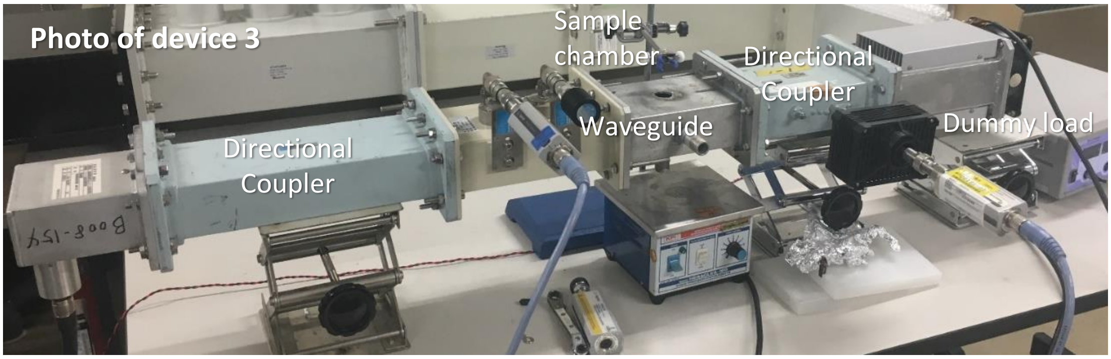

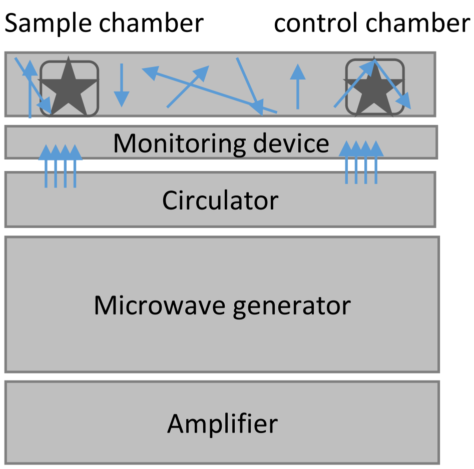

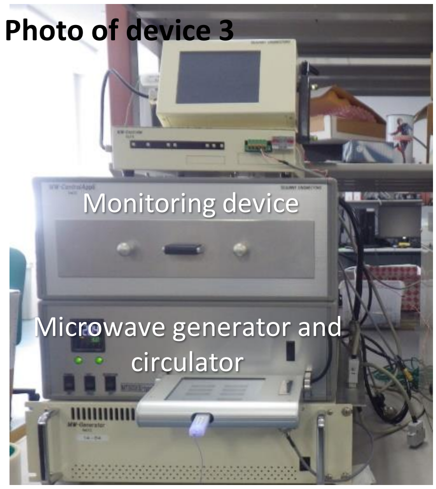

Figure S3. Multiway travelling wave irradiation, Device 3, with reflection. Blue arrows indicate the microwave route. The grey star indicates the sample insects.

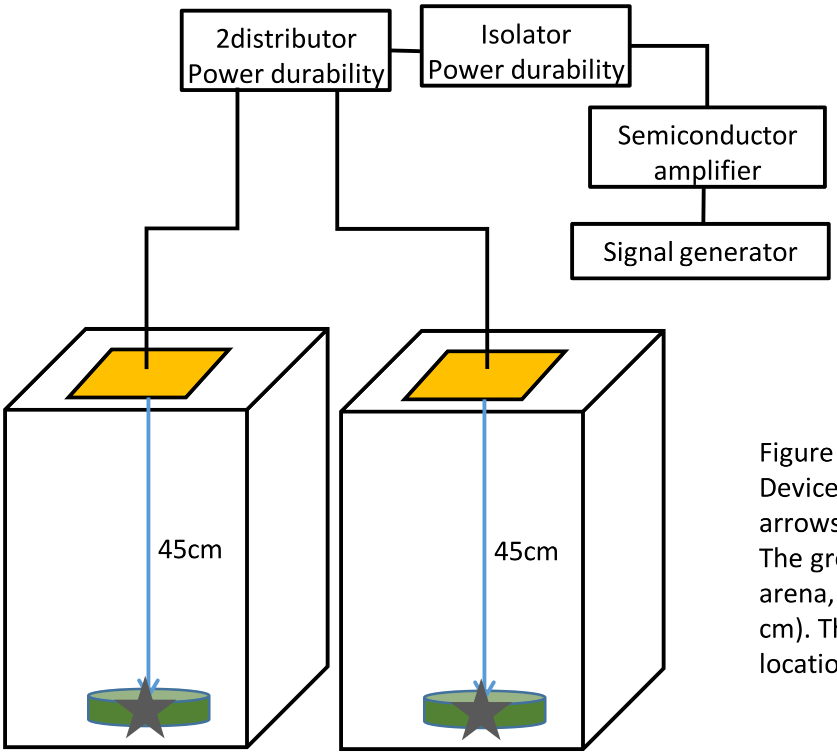

Figure S4. Traveling wave irradiation, Device 4, for the behavioral test. Blue arrows indicate the microwave route. The green part indicates the sample arena, polystyrene-petri dishes (DC 9 cm). The grey star indicates the location of sample insects.

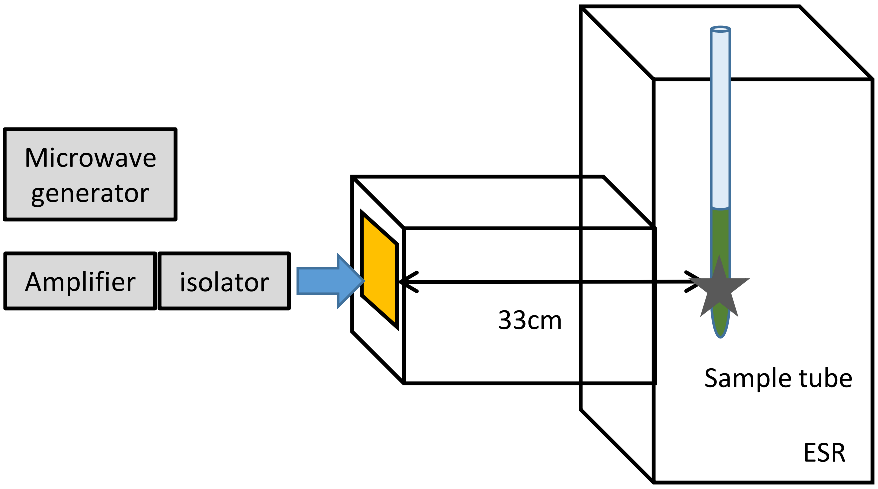

Figure S5. Travelling wave irradiation, Device 5, to give the additional irradiation during ESR recording. Portable microwave generator was set in front of the sample arena of ESR. Blue arrows indicate the microwave route. The green part indicates the sample arena. The grey star indicates the location of sample insects.

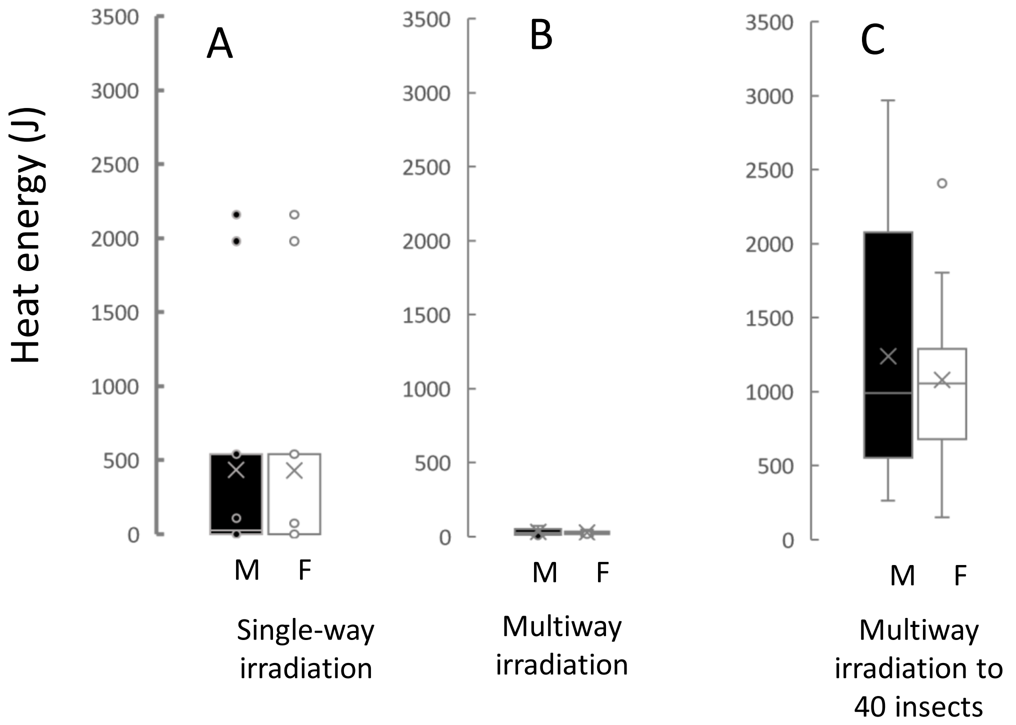

Figure S6. Energy absorption of insect body under 3 min irradiation with straight forward travelling waves. A: Energy lost by passing through one insect body under irradiation without reflection (n = 20). B: Energy lost by passing through one insect body calculated from the amount through 40 insects in (C) (n = 10). C: Heat energy caused from irradiated microwave energy while passing through 40 insect bodies under irradiation with reflection (n=10)

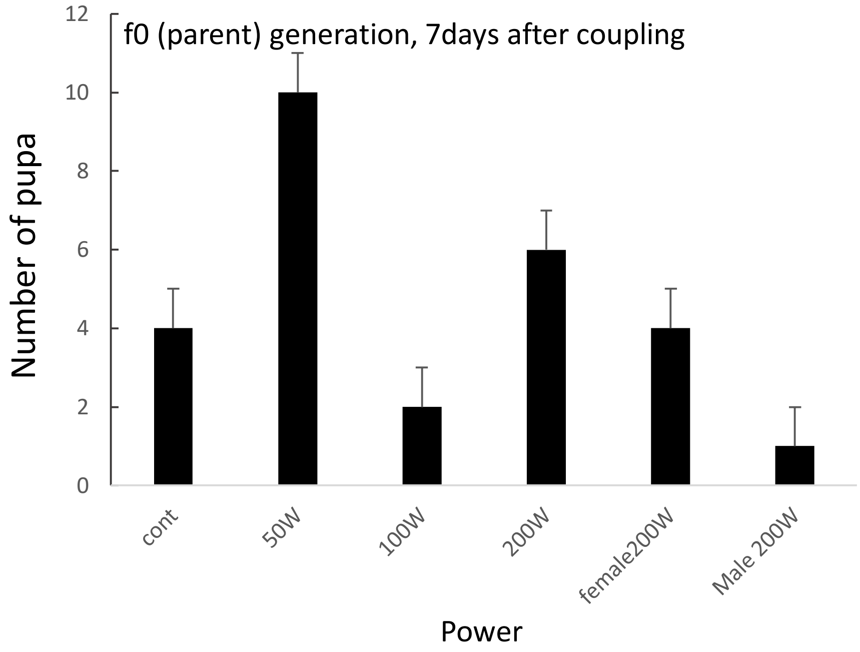

Figure S7. Effects of straightforward traveling waves on *Drosophila* fecundity. Figure shows the number of pupae from treated individuals (n=5).

Figure S8. Permittivity of the fruit flies, *D. melanogaster*.

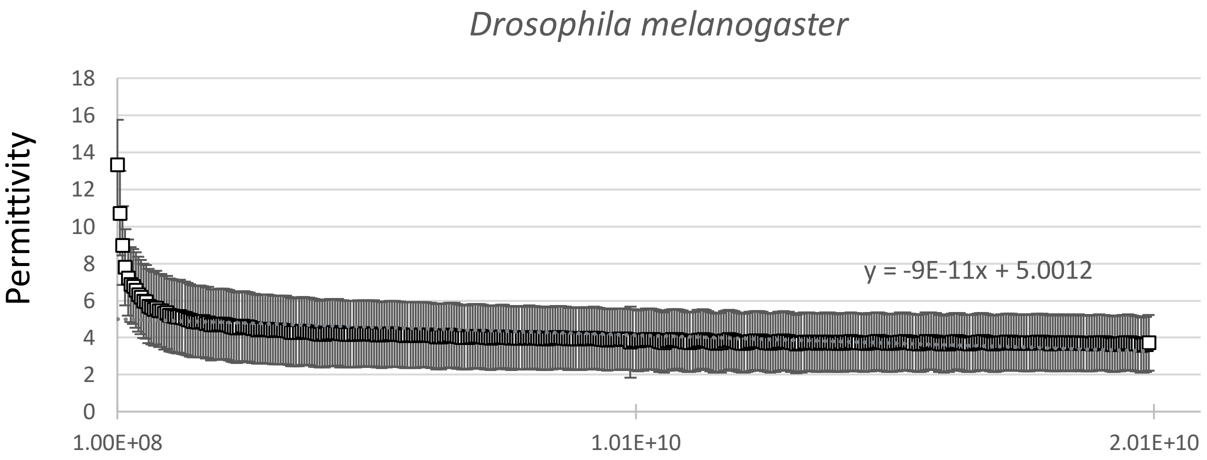

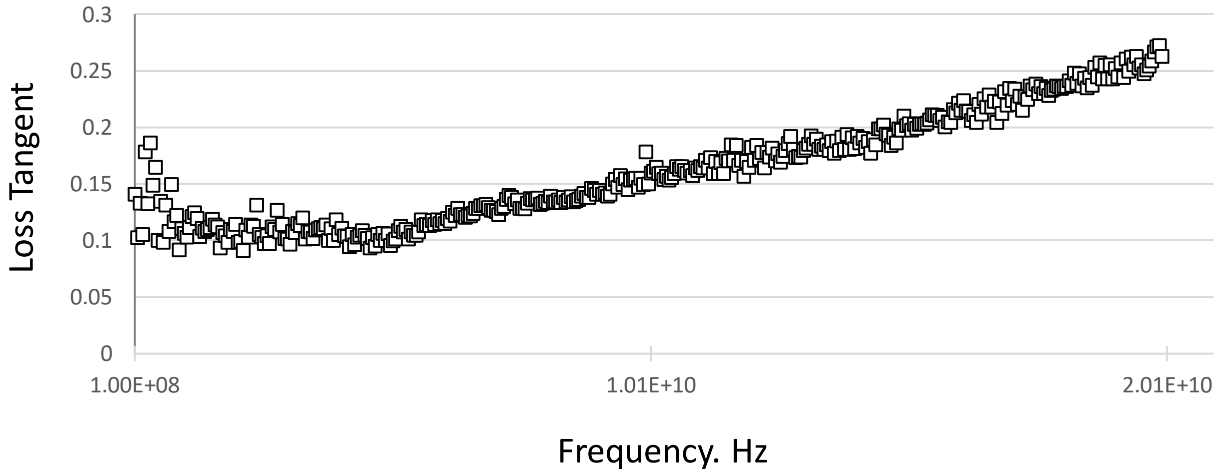

$compensation\ formura\ for\ permittivity$ (Nelson 1997)

$$\text{Permittivity } \varepsilon_\omega = \left[\frac{\epsilon^{\frac{1}{3}}+v_\omega-1}{v_\omega}\right]^3$$

ε_ω: compensation permittivity of *Drosophila*

ϵ: complex permittivity including air 4.57, v_ω: Density of *Drosophila* body 0.455g/cm^3
